# Individualized Surgery: Gamma-Probe-Guided Lymphadenectomy in Patients with Clinically Enlarged Lymph Node Metastases from Melanomas

**DOI:** 10.1245/s10434-012-2841-1

**Published:** 2013-01-12

**Authors:** Lutz Kretschmer, Carsten-Oliver Sahlmann, Pavel Bardzik, Christina Mitteldorf, Hans-Joachim Helms, Johannes Meller, Michael Peter Schön, Hans Peter Bertsch

**Affiliations:** 1 Department of Dermatology, Venereology and Allergology, Georg August University, Göttingen, Germany; 2 Department of Nuclear Medicine, Georg-August-University of Göttingen, Göttingen, Germany; 3 Department of Dermatology, Venereology and Allergology, Klinikum Hildesheim GmbH, Hildesheim, Germany; 4 Department of Medical Statistics, Georg August University of Göttingen, Göttingen, Germany

## Abstract

**Background:**

The value of a preoperative lymphoscintigraphy in melanoma patients with clinically evident regional lymph node metastases has not been studied. Therapeutic lymph node dissection (TLND) is regarded as the clinical standard, but the appropriate extent of TLND is controversial in all lymphatic basins.

**Patients and Methods:**

Of the 115 consecutive patients with surgery on palpable lymph node metastases, 34 received a pre-operative lymphoscintigraphy. Lymphatic drainage to a second nodal basin outside the clinically involved basin was found in 15 cases. In 13 patients, the ectopic tumor-draining lymph nodes were excised as in a sentinel node biopsy. The lymph nodes from the TLND specimens were postoperatively separated and classified as either radioactive or non-radioactive.

**Results:**

A total of 493 lymph nodes were examined pathologically. The largest macrometastasis maintained the ability to take up radiotracer in 77% of cases. Radioactively labeled lymph nodes carried a higher risk of being involved with metastasis. The proportions of tumor involvement for radioactive and non-radioactive lymph nodes were 44.5 and 16.9%, respectively (*P*=0.00002). Of the 13 ectopic nodal basins surgically explored, six harbored clinically occult metastases.

**Conclusion:**

In patients undergoing TLND for palpable metastases, tumor-draining lymph nodes in a second, ectopic nodal basin should be excised, because they could be affected by occult metastasis. With respect to radioactive lymph nodes situated within the nodal basin of the macrometastasis but beyond the borders of a less-radical lymphadenectomy, further studies are needed.

Lymph node metastasis is the most frequent form of first recurrence in patients with cutaneous melanoma if no lymph node surgery was performed at initial diagnosis.^1^ Regional metastases of melanomas most frequently involve the cervical, axillary or inguinal lymph node basins. Lymphatic mapping with sentinel lymphadenectomy (SLNB) has become the standard approach in treating high-risk melanoma patients with clinically unsuspicious regional lymph nodes. SLNB studies have shown that lymphatic drainage to a second or even third nodal basin is not uncommon; this happens most frequently in patients with melanomas originating on the trunk.^2^–^4^ Although melanomas located on extremities usually drain to the ipsilateral inguinal or axillary basins, additional drainage to interval nodes, iliac, popliteal or epitrochlear SLNs may be found.^5^–^7^ Since occult lymphatic metastases most often occur in the primary tumor-draining lymph nodes, there is consensus that sentinel lymph nodes (SLNs) should be excised regardless of their anatomic location.

In patients with palpably enlarged node metastases, the current standard procedure is therapeutic regional lymph node dissection (TLND) of the involved nodal basin. The value of lymphatic mapping has not yet been studied. The experience with SLNB suggests, however, that clinically occult metastasis to a second nodal basin might represent a realistic danger also for patients with enlarged nodes, especially when the primary melanoma site is suggestive for ambiguous lymphatic drainage.

Moreover, as with SLNB, the clinically unsuspicious but radioactively labeled lymph nodes within a nodal basin might carry a higher risk of metastasis, even after the formation of macrometastases. If so, the anatomic location of the radioactive nodes within a nodal basin might influence the extent of the lymph node dissection. The high morbidity and significant nodal basin recurrence rates following TLND make it necessary to pursue two aims in testing lymphatic mapping in patients with enlarged node metastases: (1) to detect all lymph nodes at risk for metastasis and (2) to avoid unnecessary extension of the node dissection. In the present analysis, we focus on these questions by reviewing 34 patients treated individually who received lymphoscintigraphy prior to excision of clinically enlarged regional lymph node metastases.

## Patients and Methods

### Patients

Between May 1998 and May 2011, 115 consecutive patients with clinically evident regional lymph node metastases from melanomas were treated at the University Medical Center in Göttingen. Of these, 25 had developed nodal recurrence following negative SLNB. There was no history of primary melanoma and no primary tumor could be located in 14 additional patients. The remaining 76 patients, who are the subject of the present study, had a known primary melanoma but no previous lymphoscintigraphy. In 29 patients, the primary melanoma was diagnosed at the same time as palpable metastases. Nodal recurrences after primary tumor excision were noted in 47 patients.

Magnetic resonance imaging of the head and neck were undertaken in patients with cervical metastases. All patients with inguinal nodal metastases received computed tomography scans in order to detect enlarged nodes in the lesser pelvis. The size of the lymphadenopathy was determined by preoperative ultrasound B-scans. In the majority of cases, fine-needle aspiration cytology was performed. In three patients who had undergone diagnostic metastasectomy, the size of the macrometastases was taken from the pathology report. Patients with clinical evidence of systemic metastases were not considered in the present study.

### Lymphatic Mapping

From November 2000, 34 selected patients received preoperative lymphoscintigraphy. In this group, two patients were actually referred for SLNB but macrometastasis >1 cm was diagnosed on preoperative ultrasound B-scans. The remaining 32 patients had clinically palpable disease.

Lymphatic mapping was deemed necessary: (1) if ambiguous lymphatic drainage from the primary melanoma site was conceivable or (2) if some kind of less radical lymph node dissection had been planned. In such cases, we aimed to excise all clinically unsuspicious but radioactive lymph nodes, even if they were situated beyond the borders of the previously designated node dissection field. All patients gave informed consent before undergoing gamma-probe-guided lymphadenectomy.

Patients with unknown primary melanoma sites as well as the false-negative cases after initially negative SLNB did not receive lymphatic mapping. Lymphatic mapping was also not carried out for patients with clinically evident metastases in two nodal basins, for patients with enlarged pelvic metastases, or for patients with grossly enlarged, fixed or matted nodes. Patients who had a primary tumor excision requiring reconstruction of the defect using skin flaps did not undergo lymphoscintigraphy. Patients with previously excised in-transit metastases were also not considered eligible for lymphatic mapping. We did include, however, patients with synchronous, surgically amenable in-transit disease. We also included three patients on whom diagnostic excision of the macrometastasis had been performed because we felt that bidirectional lymphatic drainage from the primary tumor site could still be detected.

Technically the lymphoscintigraphy did not differ from those applied on our patients with clinically unsuspicious lymph nodes.^5^,^6^ With a PICKER SX 100—a broad-view gamma camera equipped with a low-energy high-resolution collimator—preoperative dynamic lymphoscintigraphy utilizing dynamic acquisition during the first 30 min and static imaging after 1–2 h was performed on each of the patients. Approximately 18–24 h before the operation, 100 MBq of 99mTc-human albumin (Nanocoll; Nycomed Amersham Sorin) dissolved in a volume of 0.1–0.2 ml was injected into the dermis surrounding the primary melanoma or the biopsy scar. This relatively high dose was chosen to enhance the imaging process of the afferent lymphatics or deeply situated tumor-draining lymph nodes, e.g., iliac, subcostal or parasternal nodes. Static images were taken anteriorly and laterally 30 min and 2 h after injection.

### Surgical Treatment

Local excision with adequate safety margins was the standard treatment procedure of primary melanomas. Well-established standard surgical techniques of TLND were considered as standard of care (modified neck dissection, axillary dissection including nodal levels I–III with preservation of the pectoralis minor muscle, ilioinguinal dissection). Our surgical approaches of TLND have been previously described.^8^,^9^ An abdominoperineal rectum extirpation along with the enlarged paraproctic node metastases was performed on one patient.

### Less Radical Node Dissections

Generally, our approach was rather conservative in patients with increased general morbidity, with preexisting swellings of extremities or severe adiposity, with metastasis to more than one nodal basin, or in patients with preceding in-transit metastases. The latter group has been shown to have high risk of nodal basin recurrence despite thoroughly performed TLND.^8^ We distinguished between two types of less radical node dissections: (1) less radical but well-standardized dissections (selective neck dissection, dissection of axillary levels I–II only or exclusively inguinal dissection without pelvic dissection) and (2) even more limited, nonstandardized lymph node excision (LLND), which sometimes seemed to be appropriate for the reasons mentioned above. In our patients who underwent lymphatic mapping, every less radical operation included the removal of all radioactively labeled lymph nodes. During gamma-guided lymph node excision, a handheld gamma probe was used (Gamma Finder; W.O.M. World of Medicine AG, Ludwigsstadt, Germany). At the end of TLND, the borders of the node dissection area were generally checked for remaining radioactivity to ensure that all tumor-draining lymph nodes had been excised.

### Ectopic Tumor-Draining Lymph Nodes

In cases displaying bidirectional lymphatic drainage, the radioactive lymph nodes located outside the clinically involved basin were excised in the same routine manner as in an SLNB. When micrometastasis in an additional nodal basin was diagnosed, no further TLND was performed.

### Histological Analysis

Primary tumors were examined using routine histological methods. The SLNs excised from an additional nodal basin outside the clinically involved basin underwent step sections as previously described.^10^ Immunohistochemical staining was performed using the streptavidin–biotin complex method using alkaline phosphatase as the labeling enzyme and fast red chromogen as the substrate (detection kit K5005; Dako, Germany). The following antibodies were used: S-100 (clone S-100, dilution 1:3,000; Dako), HMB-45 (clone HMB45, dilution 1:200; Dako), MART-1 (clone A 103/M2-7C10/M2-9E3, dilution 1:200; Zymed, USA), and Pan-Melanoma Cocktail (clone HMB45þM2-7C10þM2-9E3þT311, dilution 1:300; Biocare Medical, USA) stained by an auto-immunostainer (Immunostar 80, Shandon Varistain 24-4, Germany).

Immediately after the surgical procedure, the lymph nodes were separated from the TLND specimen. Using the gamma probe it was possible to detect low amounts of radioactivity by touching the excised lymph nodes with the tip of the probe. Lymph nodes were considered radioactive whenever more than 4 counts were measured ex vivo in the absence of any background radiation.

Depending on their size, the lymph nodes from TLND specimens were sliced into two to four sections, each of which was embedded separately in paraffin. From each slice four microtome samples were produced and stained using hematoxylin and eosin (H&E), as well as immunohistochemical staining with anti-protein S-100 serum, MART-1, and anti-HMB-45.

### Statistical Methods

Patient data including clinical and lymphoscintigraphic parameters, as well as histopathological results were entered routinely into an electronic database. For the present analysis descriptive statistics were applied. The chi-square test was used to compare the probabilities of metastatic involvement for radioactive and nonradioactive lymph nodes. The proportions of metastatic involvement of both radioactive and nonradioactive lymph nodes were also determined for each patient, and the means were compared using the *t*-test for dependent samples. The difference in the means was characterized with a 95 % confidence interval. Analyses of survival and relapse rates were performed using Kaplan–Meier estimates. The significance level was set at *α* = 5 %.

## Results

### Patients’ Characteristics

The characteristics of the patients displaying clinically enlarged metastases who were included in this study are summarized in Table 1. The majority of the patients undergoing gamma-guided lymphadenectomy (62 %) had a primary melanoma and clinically enlarged nodal metastases at the same time, whereas the majority of the patients without lymphatic mapping (81 %) had delayed node dissection of nodal recurrences. Due to this imbalance, the patients with lymphatic mapping tended to have more aggressive primary tumors. Furthermore, our selection criteria imply a lower nodal tumor burden in the group with gamma-guided surgery, although this cannot be statistically proven through the number of pathologically involved lymph nodes.

**Table 1:** Characteristics of the patients with clinically evident lymph node metastases

	No lymphoscintigraphy	Gamma-guided lymphadenectomy	*P*
	*N* = 42	*N* = 34	(*u*-Test)
Lymphadenectomy concomitantly with primary tumor excision	8 (19 %)	21 (62 %)	0.0002
Lymphadenectomy metachronously with primary tumor excision (nodal recurrences)	34 (81 %)	13 (39 %)	
Location of macrometastasis (*N*)	Neck (3)	Neck (2)	
	Axilla (23)	Axilla (19)	
	Groin (16)	Groin (12)	
		Paraproctium (1)	
Follow-up (months)	33.7 ± 43.1	27.4 ± 23.4	0.64
Age, median (min–max) (years)	65 (18–84)	60 (30–83)	0.99
Sex, female/male	17/25	16/18	0.62
Breslow, mean ± SD (mm)	4.65 ± 5.3	5.8 ± 4.7	0.13
Breslow, median (min–max) (mm)	3.0 (0.5–30)	5.4 (0.65–23)	
Ulceration present	17 (40.5 %)	21 (63.6 %)	0.10
In-transit metastases prior to lymphadenectomy	11 (26 %)	6 (18 %)	0.56
Mean number of positive lymph nodes	4.9 ± 5.6	3.8 ± 2.9	0.84
Mean number of lymph nodes excised	19.8 ± 20.4	14.5 ± 7.2	0.37

In the group without lymphoscintigraphy, six patients received some type of less radical lymphadenectomy: One patient had selective neck dissection; two patients with bilateral axillary metastases received axillary LLNDs (sparing the level III nodes, the lateral nodes, and the lymphatic on the ventral side of the major vessels); three patients received an inguinal lymphadenectomy without pelvic dissection.

Of the 34 patients with gamma-guided lymphadenectomy, 26 received a standard TLND (8 of them had lymphatic drainage to ectopic SLNs). The remaining eight patients received some form of less radical lymphadenectomy: One patient had axillary dissection of the first two levels only; three patients received an exclusively inguinal lymphadenectomy, and five had nonstandardized LLNDs. Of the eight patients with less radical procedures, seven had ectopic SLNs. Some patients had more than one reason for restricting the procedure, such as in-transit metastases, significantly increased general morbidity, or bilateral node excision. Importantly, all radioactive lymph nodes were excised in all less radical procedures. The percentages of patients undergoing less radical procedures in the groups with and without lymphatic mapping were 24 and 14 %, respectively. As a result, the mean number of excised lymph nodes was lower in the lymphatic mapping group (14 versus 20 nodes).

### Analysis of the Lymphatic Mapping Group

The greatest diameter of the largest metastasis ranged from 1.1 to 6.5 cm (median 3.0 cm). Lymphoscintigraphy detected at least one radioactive node in 30 of the 34 clinically involved basins. All but one patient displayed at least one radioactive lymph node; the average was five radioactive nodes per patient. In the surgical specimens, the largest macrometastasis was radioactive in 24 of the 31 cases available for analysis. The median number of excised radioactive lymph nodes was 5 (range 0–15).

Most of the primary tumor sites were suggestive for bivalent lymphatic drainage; 16 were situated near the midline of the body, whereas 15 were located in a border region between two ipsilateral nodal basins. Lymphatic drainage to a second (ectopic) nodal basin included the axilla (*n* = 8), the parasternal nodes (*n* = 1), the groin (*n* = 4), the lesser pelvis (in the absence of drainage to the ipsilateral groin, *n* = 1), and the popliteal fossa (*n* = 1). Of the 15 additional nodal basins detected through lymphoscintigraphy, 14 were surgically explored. The corresponding SLNs were successfully excised in 13 patients (Table 2).

**Table 2:** Characteristics of the patients with lymphatic drainage to ectopic nodal basins

Patient	Location of the primary melanoma	Clinically involved basin	Surgical treatment of the involved basin	Second nodal basin	Surgical treatment of the ectopic basin	Pathologic status of the ectopic basin
1	Anus/rectum midlinea	Paraproctium	Abdominoperineal resection	Right groin	SLNB	Positive
2	Back midline	Groin	Inguinal TLND	Contralat. groin	SLNB	Positive
3	Back midline	Groin	Ilioinguinal TLND	Ipsilat. axilla	SLNB	Negative
4	Right back	Axilla	Axillary TLND	Contralat. axilla	SLNB	Positive
5	Back midline	Axilla	Axillary LLND	Contralat. axilla	SLNB	Positive
6	Introitus urethras	Groin	Inguinal TLND plus ipsilateral iliac SLNB	Contralat. groin	SLNB	Negative
7	Left little toe	Groin	Ilioinguinal TLND	Popliteal	SLN Not Found	
8	Back midline	Axilla	Axillary TLND	Contralat. axilla	SLNB	Positive
9	Left epigastrium	Axilla	Axillary TLND	Parasternal	Not Exposed	
10	Back midline	Back^a^	Excision with safety margin, ipsilat. axillary LLND	Contralat. back^b^	SLNB	Negative
11	Right shoulder	Supraclavicular modes	Supraclavicular LLND	Ipsilat. axilla	SLNB	Positive
12	Lumbar midline	Groin	Ilioinguinal TLND	Contralat. groin	SLNB	Negative
13	Sternum midline	Axilla	Axillary TLND	Contralat. axilla	SLNB	Negative
14	Umbilicus midline	Groin	Inguinal TLND	Contralat. Iliac nodes	SLNB	Negative
15	Back midline	Axilla	Axillary TLND^c^	Contralat. axilla	SLNB	Negative

*TLND* therapeutic lymph node dissection, *LLND* less extended, atypical lymph node excision including macrometastasis and radioactive nodes, *contralat*. contralateral, *ipsilat.* ipsilateral, *SLNB* sentinel lymph node biopsy

^a^11 o’clock in the “lithotomy position”

^b^Triangular intermuscular space

^c^Axillary dissection of levels I–II only

### Pathological Findings

SLNs situated in a separate (ectopic) nodal basin were involved with micrometastasis in six cases (four patients with simultaneous excision of primary melanoma and lymph node metastases and two patients with delayed lymph node dissection for a palpable recurrence).

The metastatic disease was restricted to the radioactive nodes in 15 of the 29 patients undergoing standardized TLND. Patients with metastases exclusively within radioactive nodes had a significantly lower number of lymph node metastases as compared with patients with negative nonradioactive nodes (2.9 ± 1.9 versus 5.0 ± 4.3 node metastases, *P* = 0.03). In five patients (one of whom did not display any radioactive nodes), the metastasis was restricted to the nonradioactive nodes. Overall, we excised 172 radioactive lymph nodes; 71 (41.2 %) were determined to be pathologically positive. Of the 321 nonradioactive lymph nodes excised, 55 (17.1 %) were pathologically positive. The proportion of tumor involvement for radioactive and nonradioactive lymph nodes was also calculated for each patient. The resulting mean probabilities were 44.5 and 16.9 %, respectively [*P* = 0.00002, difference 27.6 % (95 % confidence interval 12.3–43.1 %)]. Thus, the radioactively labeled nodes carried a significantly higher risk of being tumor-involved.

### Follow-Up

**Fig. 1:**
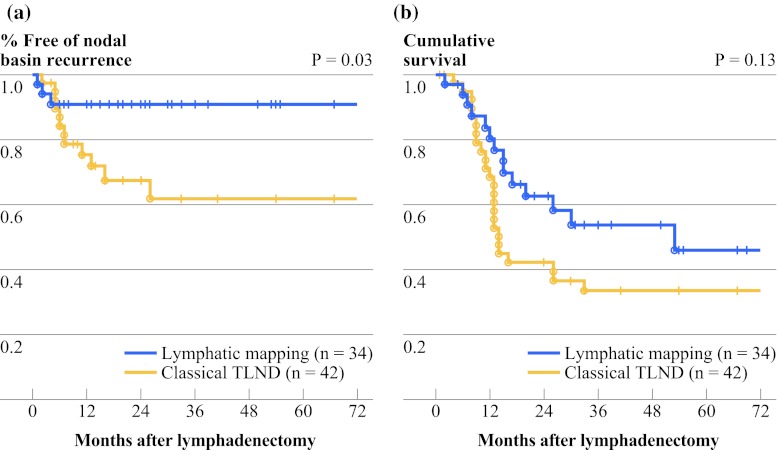
Local recurrence rates (including relapses after generalized metastases) (**a**) and survival rates (**b**) for patients with clinically enlarged regional lymph node metastases who had either gamma-guided or classical therapeutic lymph node dissection (TLND). The estimated local failure- and survival rates for all 77 patients with palpable metastases were 25 and 39 %, respectively

Local recurrence after lymphadenectomy was defined as any evidence of recurrent disease within the surgical basin harboring the macrometastasis, including relapses after generalized metastasis. The recurrence and survival rates for the patients with and without lymphoscintigraphy are shown in Fig. 1. The two groups are not directly comparable because our selection criteria appeared to favor a higher nodal tumor burden in the control group. However, a local recurrence rate of about 10 % in the lymphoscintigraphy group seems to be an acceptable result for a group with exclusively palpable nodes and a mean Breslow thickness of 5.8 mm. Of the patients who underwent some kind of less radical gamma-guided dissection, two recurred within the node dissection field. One of them had initially received excision of in-transit metastases and LLND of cervical macrometastases. In this patient, the disease recurred with the same metastasis pattern. The second patient had a superficial inguinal node dissection plus excision of contralateral pelvic SLNs. This disease recurred within the scar of the inguinal dissection simultaneously with hepatic metastasis. We did not observe any recurrences in any of the 13 ectopic basins. However, one patient diagnosed with initial lymphatic drainage to only one axilla showed recurrence in the contralateral axilla. Thus, in the group with gamma-guided lymphadenectomy, 20.6 % of the patients showed metastasis to more than one nodal basin.

In the group without lymphoscintigraphy, 9.5 % of the patients showed metastasis to two different nodal sites: One patient had bilateral cervical metastasis; two patients had bilateral axillary metastases. A further patient with a primary melanoma of the calf and enlarged inguinal and iliac metastases developed an isolated recurrence in the fossa poplitea.

## Discussion

We have demonstrated that the concept of an “orderly progression” of melanoma nodal metastases maintains its validity for the majority of patients with clinically enlarged nodal metastases.^11^ Metastasis to lymph nodes outside a clinically involved basin can be detected by lymphoscintigraphy and gamma-probe-guided lymph node dissection. This is an observation that has not been mentioned in any previous studies. Another observation especially worthy of note is that the radioactive lymph nodes within a clinically involved nodal basin carried a significantly higher risk of being involved with metastases.

The mean number of radioactive lymph nodes was five per patient, which appears to be high in comparison with SLNB. Here we confirm that a considerable proportion of the enlarged node metastases maintain the capacity to take up radiotracer.^12^ The excised macrometastases were, however, often far less radioactive when compared with the SLNs of patients with clinically unsuspicious nodes, making them easy to miss by lymphoscintigraphy.

Our observations underscore the need for a more individualized approach in lymph node surgery in patients with enlarged regional lymph node metastases. In fact, the surgical treatment of clinically enlarged node metastasis has not changed in a noteworthy manner for many years. A TLND is usually performed, in which the macrometastasis and the neighboring unsuspicious lymph nodes constituting a nodal basin or a level of a nodal basin are removed. While less than 30 % of completion lymph node dissections are tumor-positive after excision of a micrometastasis in a sentinel lymph node (SLN), this proportion rises to 55–75 % following the diagnostic excision of a clinically enlarged metastatic node.^13^–^18^ Moreover, as compared with SLNB and completion lymph node dissection, performed at an early stage, TLND for clinically enlarged metastases has yielded a significantly higher number of affected lymph nodes.^19^,^20^ In our study, the nodal disease was restricted to the radioactive nodes in only 52 % of the patients with radical procedures. It also has to be considered that local failure rates after excision of palpable node metastases are unsatisfactory.^8^,^9^,^21^ These observations support the present standard of performing radical TLNDs for clinically enlarged node metastases. Still, there are three main goals: cure, regional tumor control, and staging. Long-term survival can be achieved in about 29–52 % (39 % in the present study).^22^

One especially important problem is, however, that radical lymph node dissection carries risks of considerable morbidity, which may substantially affect a patient’s quality of life. From this point of view, an ideal lymphadenectomy should include all metastases but only a minimum of tumor-free lymph nodes. So far, the SLN concept has enhanced research in this direction only in patients with clinically occult metastases.^23^,^24^

Up until now, the appropriate extent of a TLND has remained controversial for all lymphatic basins.^25^,^26^ For the treatment of neck metastases, functional neck dissection or selective neck dissections are presently replacing radical neck dissection. However, almost a quarter of head and neck melanomas metastasize outside clinically predicted neck levels.^27^ In the axilla, level I, II, and III dissection is most commonly performed, although some include level III only when suspicious nodes are present.^18^ Considering the poor prognosis of patients with iliac metastases and the increased rate of lymphedema after iliac clearance, some surgeons advise to exclusively perform a superficial inguinal node dissection when inguinal nodes are palpably enlarged.^28^,^29^ In some instances, an even less radical, atypical bloc dissection may be performed in selected patients, mostly because of significant general morbidity. A limited bloc dissection may also be considered adequate if surgically treatable locoregional cutaneous metastases are present. It is an unfortunate truth that the recurrence rates of nodal basin metastases are high in such patients despite thorough TLND.^8^,^9^ Metastasis to a second nodal basin might be a further indication for a less radical approach, in order to avoid increased postoperative morbidity. This is the reason why we did not perform a second TLND after the excision of ectopic SLN metastasis. Fortunately, we did not observe recurrences in an ectopic nodal basin.

Clearly, even radical TLND loses its efficacy if occult nodal metastasis is present outside the node dissection field. It therefore seems advisable to remove all radioactive nodes, i.e., the nodes that face a higher risk of being tumor-involved. In the present study, the overall number of excised nodes was higher in the patients with classical TLND than in the patients with lymphatic mapping (20 versus 14 nodes). Nevertheless, regional control did not appear to be compromised after gamma-guided lymphadenectomy (Fig. 1).

The demonstration of occult, ectopic lymph node metastases in this study is the most convincing argument in favor of a more individual approach in lymph node surgery of enlarged regional lymph node metastases. A considerable proportion of the patients suspected to have ambiguous lymphatic drainage from their primary tumor sites did indeed display tumor-draining lymph nodes outside the clinically involved nodal basin. Of the 13 additional nodal sites that were explored successfully, 6 (46 %) were involved with occult metastasis. It is noteworthy that we found lymph node metastases outside the clinically involved basin, both in patients with clinically enlarged nodal metastases at initial diagnosis, as well as in patients with nodal recurrences.

Unfortunately, for some patients, lymphatic mapping does not seem to be an option: Grossly enlarged or matted nodes seem to be unsuitable. Moreover, flap reconstruction at the primary tumor site may lead to inaccurate results of lymphoscintigraphy.^30^ The original lymphatic drainage pathway from the primary tumor site can also be destroyed following the excision of in-transit metastases or after a false-negative SLNB. We did include, however, three patients on whom a diagnostic excision of the macrometastasis had been performed because we felt that bidirectional lymphatic drainage from the primary tumor site could still be detected.

A gamma-guided lymphadenectomy might be helpful to avoid missing the radioactive lymph nodes, i.e., the nodes with the highest risk of metastatic involvement. In patients with selective neck dissections, superficial inguinal dissection, or other types of less radical lymph node excision, radioactively labeled lymph nodes may be situated beyond the borders of the dissection. Theoretically, such radioactive nodes might be primary tumor-draining nodes but also second-echelon nodes or nodes receiving lymph from anastomoses formed due to metastatic blockage of the original lymphatic drainage pathways. Further studies on the impact of these nodes are needed. Our results clearly indicate that lymphatic mapping should not be withheld from patients with clinically enlarged node metastases who may display ambiguous lymphatic drainage from their primary tumor site. As shown in the present study, tumor-draining lymph nodes in ectopic nodal basins can be affected by occult metastases. Performing only a standard TLND on the patients concerned might negatively affect staging, local tumor control or even survival.
